# *BRAF*突变在非小细胞肺癌中的研究进展

**DOI:** 10.3779/j.issn.1009-3419.2024.101.01

**Published:** 2024-01-20

**Authors:** Libian DENG, Yaxian YANG, Jian HUANG

**Affiliations:** ^1^524002 湛江，广东医科大学附属第二医院病理科（邓李变）; ^1^Department of Pathology, The Second Affiliated Hospital of Guangdong Medical University, Zhanjiang 524002, China; ^2^510700 广州，广州华银康医疗集团股份有限公司（杨雅娴）; ^2^Guangzhou Huayin Health Medical Group Co., Ltd, Guangzhou 510700, China; ^3^524001 湛江，广东医科大学附属医院病理诊断与研究中心（黄剑）; ^3^Department of Pathological Diagnosis and Research Center, The Affiliated Hospital of Guangdong Medical University, Zhanjiang 524001, China

**Keywords:** 肺肿瘤, BRAF基因, 靶向治疗, Lung neoplasms, BRAF gene, Targeted therapy

## Abstract

鼠类肉瘤病毒癌基因同源物（V-Raf murine sarcoma viral oncogene homolog B, BRAF）突变是非小细胞肺癌（non-small cell lung cancer, NSCLC）的重要驱动基因之一。BRAF基因编码丝氨酸/苏氨酸蛋白激酶，BRAF突变通常导致其编码蛋白质的活化，从而导致丝裂原活化蛋白激酶激酶（mitogen-activated protein kinase kinase, MEK）信号传导途径的激活。针对BRAF突变或其下游MEK靶向药物的临床应用，为BRAF突变的NSCLC提供了更为针对性及有效的治疗。然而这些方案也存在获益持续时间短、BRAF非V600突变治疗效果差、易耐药等问题，需要新的复合治疗方案来改善。本文就BRAF基因结构特点、相关信号通路、突变类型，尤其是BRAF突变和NSCLC的临床病理联系及治疗进展等方面进行综述，为临床医生选择更有效的治疗方案提供依据。

## Abstract (en)

V-Raf murine sarcoma viral oncogene homolog B (BRAF) alteration is one of the most essential driver genes of non-small cell lung cancer (NSCLC). BRAF encodes serine/threonine protein kinases, and its mutations typically lead to protein compositional activation, thereby activating the mitogen-activated protein kinase kinase (MEK) signaling pathway. A promising new approach for the treatment of mutated BRAF and/or downstream MEK may provide customized treatment opportunities for BRAF driven NSCLC patients. However, combination therapy is necessary to overcome the difficulties such as short duration of benefit, poor therapeutic effect of non-V600 BRAF mutations and susceptibility to drug resistance. This article reviewed the progress in structural characteristics, related signaling pathways, mutation types of BRAF gene, and the clinical pathological relationship between BRAF mutations and NSCLC, as well as the therapy, in order to provide more evidences for clinical doctors to make treatment decisions.

肺癌是导致癌症相关死亡的主要原因，占全球癌症死亡人数的1/3左右。在过去大约30年间，我国肺癌的发病率和死亡率呈显著上升趋势，已演变成国内增长最快、危害最大的恶性肿瘤^[1,2]^。我国肺癌患者中，75%-90%属于非小细胞肺癌（non-small cell lung cancer, NSCLC）。晚期NSCLC通常采用铂类双联化疗，但有复发风险。

在NSCLC中，存在许多肿瘤驱动基因，如表皮生长因子受体（epidermal growth factor receptor, EGFR）、人类表皮生长因子受体2（human epidermal growth factor receptor 2, HER2）、Kirsten大鼠肉瘤病毒癌基因（Kirsten rat sarcoma viral proto oncogene, KRAS）、鼠类肉瘤病毒癌基因同源物B（V-Raf murine sarcoma viral oncogene homolog B, BRAF）、间变性淋巴瘤激酶（anaplastic lymphoma kinase, ALK）及原癌基因1受体酪氨酸激酶（c-ros oncogene 1 receptor tyrosine kinase, ROS1）等^[3]^。针对这些驱动基因的靶向治疗极大地改善了NSCLC患者的治疗效果及治疗模式，本文着重针对NSCLC中BRAF基因突变及靶向治疗进展进行综述。

## 1 BRAF基因

### 1.1 BRAF基因结构

大鼠肉瘤（rat sarcoma, RAS）/快速加速纤维肉瘤（rapidly accelerated fibrosarcoma, RAF）/丝裂原活化蛋白激酶激酶（mitogen-activated protein kinase kinase, MEK）/细胞外信号调节激酶（extracellular signal-regulated kinase, ERK）或丝裂原活化蛋白激酶（mitogen-activated protein kinase, MAPK）信号通路是一个关键的细胞内信号通路，介导细胞增殖和存活所必需的生长信号反应^[3,4]^。BRAF是一种丝氨酸/苏氨酸蛋白激酶，与ARAF和CRAF同属于RAF激酶家族，参与MAPK信号通路。

BRAF基因位于第7号染色体长臂上，由3个蛋白保守结构区（conserved regions, CR）组成，分别为RAS结合结构域（CR1）、调节结构域（CR2）和激酶结构域（CR3）^[5]^。CR1含有与Ras-GTP结合的RAS结合结构域（RAS binding domain, RBD）和富含半胱氨酸结构域（cysteine-rich domain, CRD），是RAS蛋白间互动的关键性环节^[4]^。CR2包括1个富含丝氨酸/苏氨酸的区域，该区域具有重要的抑制性磷酸化位点，可以解除RAS结合和RAF活化^[6]^。CR3位于C末端的11-18号外显子内，是蛋白激酶结构域，其磷酸化是激酶激活的必需条件^[7]^。CR3的N端部分参与ATP结合，它包含P环区域，对稳定ATP结合和维持无活性BRAF构象有重要作用^[5]^。

### 1.2 BRAF相关信号通路

在静息细胞中，RAF定位于胞浆，其N端调节区域与激酶结构域相互作用，通过14-3-3二聚体与2个磷酸化丝氨酸位点（pS365和pS729）结合，维持自身抑制的单体状态^[8,9]^。

当生长因子与细胞表面受体酪氨酸激酶（receptor tyrosine kinases, RTKs）结合时（EGFR是最早发现的RTK），受体-配体相互作用促进细胞内部RTK的二聚化、活化和自体磷酸化，活化的RTK通过磷酸酪氨酸残基结合细胞质中带有SH2或PTB结构域的蛋白，募集生长因子受体结合2（growth-factor receptor-bound 2, GRB2）和鸟嘌呤核苷酸交换因子如SOS（Son of Sevenless），促进Ras上GTP与GDP交换^[10,11]^。RAS-GTP可以直接与RAF蛋白结合，将RAF从细胞质中募集到细胞膜上，使RAF成为一种活性激酶。随后，活化的RAF对其下游底物（即MEK和ERK）进行一系列磷酸化反应，启动经典MAPK途径的活化^[12,13]^（图1）。

**图 1:**
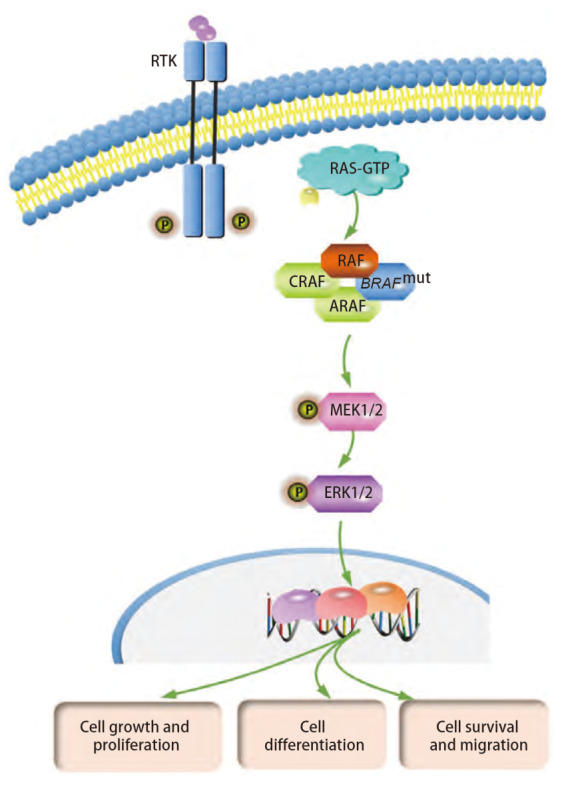
MAPK信号通路

### 1.3 实体瘤中的BRAF基因突变

RAS-RAF-MEK-ERK通路的失调与人类癌症的发生有关，基因变异造成失调的MAPK信号通路通过多种机制导致癌症的发生和发展，如促进肿瘤增殖、存活、侵袭、转移、细胞外间质降解和血管生成等。2002年，首次在黑色素瘤中详细说明了发生在BRAF基因V600位点（最初错误描述在V599位置）的点突变是致癌突变，并将其鉴定为癌基因和潜在的治疗靶点^[14]^。

研究^[15]^表明BRAF突变发生在多达8%的人类癌症中，包括黑色素瘤、结直肠癌、胶质瘤、甲状腺癌、NSCLC、胆管癌和多种血液系统恶性肿瘤。40%-60%的黑色素瘤患者发生BRAF突变，与BRAF 野生型黑色素瘤患者相比，BRAF 突变的黑色素瘤患者更年轻，且更有可能转移到大脑^[16]^。在转移性结直肠癌（metastatic colorectal cancer, mCRC）中，BRAF突变发生率为5%-10%^[17]^，然而，挪威的一项研究^[18]^表示这个数字可能被低估，BRAF基因突变在mCRC发生率可高达21%。间变性甲状腺癌（anaplastic thyroid cancer, ATC）是一种罕见的甲状腺肿瘤，BRAF V600E突变在近40%的ATC中鉴定为驱动癌基因^[19]^。最近的研究^[20⇓-22]^表明，BRAF变异也是中枢神经系统肿瘤、胆管癌的常见驱动变异。

### 1.4 BRAF基因突变类型

BRAF V600E是最常见且最著名的BRAF突变，15号外显子核苷酸T1799处的胸苷转换为腺苷，从而有效地在BRAF激酶结构域密码子600处用谷氨酸取代缬氨酸（V600E）。BRAF基因变异大多数发生在V600密码子附近的激活环（A环）或464-469残基处的磷酸结合环（P环），并可分为3种亚型。I类亚型为组成型活性RAS非依赖性单体（BRAF V600E/K/D/R），这类变异能够引起强烈的BRAF激酶活化^[23]^。II类突变形成活性RAS非依赖性二聚体，具有高或中等BRAF激酶活性，这类突变涉及600位点以外的密码子（例如：K601E、L597V/Q/R、G469V/S/R/E/A、G464V）。III类为RAS依赖性二聚体，由于激酶活性受损，需要通过RAS或RTK激活额外的上游信号来扩大ERK信号传导，这会导致致癌MAPK超活化（例如：G596R、D594Y/N/G/E、N581Y/S/I、G466V/L/E/A、D287Y）。因此，III类变异通常与MAPK途径上游的其他基因突变一起发生（例如：NF1）。II类和III类变异统称为非V600突变^[24]^。

除了15号外显子的突变，一些NSCLC患者携带BRAF 11号外显子的变异，这些突变目前并非药物作用位点^[25]^。

## 2 BRAF基因突变与NSCLC关系

### 2.1 BRAF突变在NSCLC中的流行病学与病理特征

BRAF 基因突变在NSCLC中的发生率为1.5%-4%，主要发生于肺腺癌中。在中国人群中BRAF突变频率为2.26%（5/221）^[26]^，韩国患者中BRAF突变频率为1.3%（5/378）^[27]^。在英国的大型队列研究^[28]^中，BRAF突变频率为3.44%（185/5384），而在德国队列^[29]^中，BRAF突变频率为2.34%，BRAF突变频率在不同人群中相差不大。癌症基因组图谱（The Cancer Genome Atlas, TCGA）项目对178例肺鳞癌的综合基因组评估显示BRAF变异发生频率为3%^[30]^，但肺鳞癌中的BRAF突变仍然是罕见的^[31,32]^。在NSCLC中，BRAF基因突变中最常见的是BRAF V600E，变异发生频率为1%-2%。吸烟的肺癌患者中，BRAF通常为非V600突变，而不吸烟的NSCLC患者更可能携带V600突变^[33]^。BRAF V600E突变在男性和女性中发生的频率相同，但在老年患者（年龄>60岁）中更为常见^[34]^。有研究^[35]^表明，BRAF突变NSCLC患者的淋巴结转移发生率高于非BRAF突变NSCLC患者。

携带BRAF V600E突变的NSCLC多为非黏液性腺癌，显示完全或部分微乳头状生长模式^[36]^ ，而具有BRAF非V600E突变的NSCLC可能显示各种形态，包括黏液成分^[7]^。另外，BRAF突变型NSCLC中程序性细胞死亡配体1（programmed cell death ligand 1, PD-L1）的表达略高于野生型^[37,38]^，但也有研究^[39]^表示两者之间的PD-L1表达并无差异。在BRAF突变的NSCLC中肿瘤突变负荷（tumor mutational burden, TMB）较高，但BRAF突变不影响微卫星不稳定性（microsatellite instability, MSI）和间质环境中的免疫细胞浸润数量^[39]^。

### 2.2 NSCLC中BRAF与其他驱动基因的并存状态

BRAF突变的NSCLC患者可能同时发生其他驱动基因突变，并且在BRAF V600E和非V600E病例中共突变的数量和类型具有差异。一项大队列研究^[40]^表明，SMAD4和PIK3CA的变异与BRAF V600E相关（P<0.01），而KEAP1、NF1、MET、RICTOR、KRAS、MYC、STK11和TP53的变异更频繁地发生在BRAF非V600E突变肿瘤中（P<0.05）。同时发生的RAS和BRAF V600突变极为罕见，研究^[41]^表明与BRAF V600突变相比，携带BRAF非V600突变的患者更可能发生和RAS的共突变（P≤0.001）。

大约3%的NSCLC患者中，出现原发性EGFR和BRAF V600E共突变，这将导致患者对奥希替尼（Osimertinib）的耐药，少数情况是抗EGFR-酪氨酸激酶抑制剂（tyrosine kinase inhibitors, TKIs）治疗后的获得性突变，然而目前对这类获得性BRAF突变的发生机制尚不清楚^[42,43]^。

### 2.3 BRAF与NSCLC预后

BRAF基因突变对NSCLC的预后意义尚不清楚。目前小型回顾性研究^[44]^报告了相互矛盾的结果，部分原因可能是BRAF突变的发生率较低。一项17,664例NSCLC患者的分析^[45]^表示，BRAF突变患者对一线化疗的客观缓解率（objective response rate, ORR）低于完全野生型人群（23% vs 32%），但观察到中位无进展生存期（progression-free survival, PFS）和中位总生存期（overall survival, OS）没有差异（BRAF突变型 vs 野生型：中位PFS为7.5 vs 7.1个月；中位OS为13.8 vs 11.8个月）。

一项研究^[41]^表明，236例被诊断为BRAF突变的NSCLC的患者中，有69例（29%）在诊断时发现了脑转移，对其进行回顾性分析发现，与I类变异相比，II或III类突变患者更可能发生脑转移（P≤0.01）。总体来说，BRAF在NSCLC预后影响方面的相关研究结果仍存在分歧，需要更多的大样本研究来提供解决方案。

## 3 BRAF突变治疗策略

### 3.1 BRAF突变的NSCLC对化疗不敏感

化疗是NSCLC常用的治疗策略之一，而BRAF突变的NSCLC从化疗获益有限。既往研究^[46]^发现，BRAF突变与BRAF野生型的NSCLC对铂类联合化疗的临床结果在PFS和OS方面无显著差异，表明BRAF突变与化疗敏感性无关。而BRAF V600E突变的NSCLC患者铂类联合化疗比非V600E突变的患者具有更短的PFS（4.1 vs 8.9个月），表明BRAF V600E突变患者与BRAF野生型相比预后较差。来自法国的一项研究^[47]^表明，以紫杉醇为基础的化疗在一线治疗方案中显示最差的PFS，且化疗方案与预后无关。虽然使用标准化疗或其他化疗的BRAF突变NSCLC的临床结果证据有限，但目前的研究结果表明BRAF突变的NSCLC对化疗不敏感。

### 3.2 BRAF突变的NSCLC对免疫治疗疗效有限

免疫检查点抑制剂（immune checkpoint inhibitors, ICIs）治疗是另一种选择。有研究^[37]^报道BRAF突变的NSCLC高表达PD-L1，所以BRAF突变型患者具有ICIs获益的可能。但有研究^[48]^表明，BRAF V600突变和BRAF非V600突变的NSCLC患者进行ICIs治疗后中位PFS分别为5.3和4.9个月，6个月PFS率分别为48.0%和46.1%，12个月PFS率分别为39.1%和24.6%。该项研究中，BRAF V600和非V600队列的有效率分别为26%和35%，另一项研究^[38]^也表示ICIs治疗在BRAF突变的NSCLC中疗效有限。

然而，有报道^[49]^发现1例62岁女性不吸烟NSCLC患者伴有高PD-L1表达和BRAF V600E突变，使用阿替利珠单抗（Atezolizumab）联合铂类化疗的PFS为20个月，表明联合免疫疗法可能是BRAF V600E突变不吸烟NSCLC伴有高PD-L1表达的一种治疗选择。需要进一步的临床试验来探究其疗效。

### 3.3 靶向治疗

现行的国际与国内指南规定，晚期NSCLC患者应至少进行EGFR和BRAF突变以及ALK/ROS1重排的检测，来确定正确的治疗策略^[50]^。目前批准的BRAF驱动肺癌靶向抑制剂包括达拉非尼（Dabrafenib）和维罗非尼（Vemurafenib），它们对由活性单体（即I类）突变体（主要是密码子V600E突变体）驱动的RAS非依赖性信号传导有效。84例NSCLC患者II期临床试验^[51]^结果表明：达拉非尼对BRAF V600E阳性的NSCLC治疗有效，总体反应率为33%（95%CI: 23%-45%）。VE-BASKET研究^[52]^表明，62例BRAF V600E突变型NSCLC患者接受维罗非尼治疗的ORR为37.1%（95%CI: 25.2%-50.3%），未经治疗的患者ORR为37.5%（95%CI: 8.5%-75.5%），既往接受过治疗的患者为37.0%（95%CI: 24.3%-51.3%）。中位PFS为6.5个月（95%CI: 5.2-9.0），中位OS为15.4个月（95%CI: 9.6-22.8）。由于单一BRAF抑制剂的活性有限，研究者开始探索联合治疗。

NCT01336634试验^[53]^评估了达拉非尼联合曲美替尼（Trametinib）治疗既往未经治疗的BRAF V600E突变型转移性NSCLC患者的活性和安全性。试验结果表明，既往未接受治疗的患者（n=36）的ORR为64%（95%CI: 46%-79%），中位缓解时间（duration of response, DOR）为15.2个月（95%CI: 7.8-23.5），中位PFS为14.6个月（95%CI: 7.0-22.1）。这项试验也表明，在接受达拉非尼单药治疗或联合治疗后，疾病进展时间（time to progression, TTP）与进展后生存时间（post-progression survival, PPS）持续时间显著延长^[54]^。随访结果表明，达拉非尼联合曲美替尼治疗具有实质性和持久的临床益处（既往经治疗患者的中位OS为18.2个月，初治患者中位OS为17.3个月），不论患者既往是否经过治疗，均具有可控的安全性^[55]^。基于这些良好的结果，2022年3月，双靶治疗（达拉非尼+曲美替尼）在中国获批用于治疗BRAF V600E突变NSCLC患者。2023年中国临床肿瘤学会（Chinese Society of Clinical Oncology, CSCO）指南^[56]^将双靶治疗作为BRAF V600E晚期NSCLC一线治疗I级推荐。

美国食品药品监督管理局（Food and Drug Administration, FDA）近日批准康奈非尼（Encorafenib）联合贝美替尼（Binimetinib）治疗BRAF V600E突变转移性NSCLC。II期临床试验^[57]^报道了BRAF V600E突变型NSCLC中未治疗队列（n=59）中，ORR为75%（95%CI: 62%-85%），中位DOR和中位PFS不可估计（not estimated, NE）。而在既往治疗队列（n=39）中，ORR为46%（95%CI: 30%-63%），中位DOR为16.7个月（95%CI: 7.4-NE），中位PFS为9.3个月（95%CI: 6.2-NE）。这为BRAF V600E突变转移性NSCLC的成年患者提供了一种新的个性化治疗选择。

### 3.4 BRAF靶向治疗的副作用

达拉非尼联合曲美替尼治疗对多种BRAF突变肿瘤有临床益处，但在治疗期间可观察到常见的不良事件，如发热、疲劳和恶心以及一系列皮肤病、眼睛毒性和出血事件。NCT01336634试验^[58]^中57例患者中有32例（16%）报告有严重不良事件，包括发热（16%）、贫血（5%）、精神状态混乱（4%）、食欲下降（4%）、咯血（4%）、高钙血症（4%）、恶心（4%）及皮肤鳞状细胞癌（4%）。另一项研究^[59]^表明，使用达拉非尼联合曲美替尼治疗的患者，61.3%出现发热，5.7%出现3/4级发热，15.6%出现方案定义的严重发热事件，发热通常在治疗早期发生，并随着治疗时间的延长而减少，剂量中断时可以控制。对于严重复发性发热患者，使用皮质类固醇是一种预防策略，以减少不必要的治疗中断^[60]^。

### 3.5 BRAF突变治疗耐药机制及应对策略

尽管BRAF抑制剂在BRAF突变型NSCLC患者中显示出临床活性，但由于MAPK通路的重新激活，大多数患者对这些药物产生了耐药性。BRAF靶向治疗后BRAF本身的融合、扩增和BRAF上游激活突变[如病毒致癌基因同源物（neuroblastoma RAS viral oncogene homolog, NRAS）或KRAS突变]、下游MAPK通路改变（如MEK1/2突变或ERK突变）、磷脂酰肌醇3激酶/蛋白激酶B/哺乳动物雷帕霉素靶蛋白（phosphatidylinositol-3-kinase/protein kinase B/mammalian target of rapamycin, PI3K/AKT/mTOR）等旁路活化以及受体酪氨酸激酶（例如：EGFR、PDGFRβ、MET、ERBB3、IGFR1）的表达增加，都会导致BRAF靶向抑制剂的耐药性^[61]^。

双靶治疗通过阻断ERK信号传导，在BRAF抑制剂中加入MEK抑制剂可以通过阻断ERK信号来克服或延缓BRAF抑制剂获得性抵抗力的发展。然而研究^[62]^表明，使用BRAF抑制剂或达拉菲尼和曲美替尼双重阻断后的激活突变如KRAS或NRAS也是引起耐药的原因。NRAS上调可能促进RAF的二聚化，这将导致ERK信号传导对药物不敏感，导致肿瘤抗药性^[63]^。达拉非尼和曲美替尼的联合治疗用于治疗BRAF V600突变型NSCLC中，EGFR表达上调形成了对双靶治疗（BRAF抑制剂+MEK抑制剂或EGFR抑制剂）的抗性，研究^[64]^显示使用三联靶向治疗（BRAF抑制剂+MEK抑制剂+EGFR抑制剂）可恢复细胞对治疗的敏感性。

基因进化的多样性和随机性提示我们必须对可能发生的突变进行全面分析，以确认患者的获得耐药性，并指导科学界设计针对特定耐药性的临床试验。

### 3.6 BRAF非V600E突变疗法研究

在肺癌患者中，大约70%的BRAF突变是非V600E突变，然而，目前还没有针对携带BRAF非V600E突变的患者的靶向治疗。II类突变体G469V和G469A，通过形成非RAS依赖的二聚体激活信号传导，而达拉非尼和维罗非尼只抑制二聚体中的一个原体，所以对这些II类突变体无效^[25]^。PLX8394破坏BRAF同源二聚体和BRAF/CRAF异源二聚体的形成，但不破坏CRAF同源二聚体，表明它可能比II型抑制剂具有更广泛的治疗指数^[65,66]^。最近的模型实验^[65]^表明，一些EGFR-TKIs，如吉非替尼、阿法替尼和奥希替尼，可以通过直接抑制BRAF G469V突变体来抑制肺癌生长，临床上批准的EGFR-TKIs或可用于治疗携带BRAF G469V突变的NSCLC患者。这项研究给我们的启示是，虽然特异性蛋白激酶抑制剂是癌症治疗的主要策略，但一些激酶抑制剂的“脱靶”抑制也可能显著影响癌症治疗。

## 4 总结

BRAF突变是癌基因驱动的NSCLC中的一个新兴的靶标，BRAF基因对NSCLC的发生、发展和预后起着关键作用，帮助我们深入理解其分子病理机理有重要价值。随着肿瘤治疗步入以分子标志物为治疗靶点的个体化医疗时代，系统性地对肿瘤复杂基因突变进行分子分型显得至关重要。针对BRAF V600E原发突变靶向治疗后耐药、EGFR突变的NSCLC TKIs治疗后BRAF V600E的获得性突变及BRAF非V600E突变的治疗策略，是未来该领域重点要解决的临床难题；同时，探寻EGFR/BRAF/MEK信号通路上的其他有效抑制靶点，亦是针对BRAF突变的NSCLC临床研究重点。

## References

[1] Zheng RS, Zhang SW, Sun KX, et al. Cancer statistics in China, 2016. Zhonghua Zhongliu Zazhi, 2023, 45(3): 212-220. 36944542 10.3760/cma.j.cn112152-20220922-00647

[2] Zhu MX, Hu C, He Y. Correlation of serum tumor markers and incidence of driver gene mutations in non-small cell lung cancer. Lujun Junyi Daxue Xuebao, 2022, 44(24): 2465-2473.

[3] Nguyen-Ngoc T, Bouchaab H, Adjei AA, et al. BRAF alterations as therapeutic targets in non-small-cell lung cancer. J Thorac Oncol, 2015, 10(10): 1396-1403. doi: 10.1097/JTO.0000000000000644 26301799

[4] Singh AK, Sonawane P, Kumar A, et al. Challenges and opportunities in the crusade of BRAF inhibitors: From 2002 to 2022. ACS Omega, 2023, 8(31): 27819-27844. doi: 10.1021/acsomega.3c00332 37576670 PMC10413849

[5] Riudavets M, Cascetta P, Planchard D. Targeting BRAF-mutant non-small cell lung cancer: Current status and future directions. Lung Cancer, 2022, 169: 102-114. doi: 10.1016/j.lungcan.2022.05.014 35696864

[6] Zhao J, Luo Z. Discovery of Raf family is a milestone in deciphering the RAS-mediated intracellular signaling pathway. Int J Mol Sci, 2022, 23(9): 5158. doi: 10.3390/ijms23095158 35563547 PMC9101324

[7] Leonetti A, Facchinetti F, Rossi G, et al. BRAF in non-small cell lung cancer (NSCLC): Pickaxing another brick in the wall. Cancer Treat Rev, 2018, 66: 82-94. doi: 10.1016/j.ctrv.2018.04.006 29729495

[8] Gunderwala A, Cope N, Wang Z. Mechanism and inhibition of BRAF kinase. Curr Opin Chem Biol, 2022, 71: 102205. doi: 10.1016/j.cbpa.2022.102205 36067564 PMC10396080

[9] Martinez Fiesco JA, Durrant DE, Morrison DK, et al. Structural insights into the BRAF monomer-to-dimer transition mediated by RAS binding. Nat Commun, 2022, 13(1): 486. doi: 10.1038/s41467-022-28084-3 35078985 PMC8789793

[10] Degirmenci U, Wang M, Hu J. Targeting aberrant RAS/RAF/MEK/ERK signaling for cancer therapy. Cells, 2020, 9(1): 198. doi: 10.3390/cells9010198 31941155 PMC7017232

[11] Ullah R, Yin Q, Snell AH, et al. RAF-MEK-ERK pathway in cancer evolution and treatment. Semin Cancer Biol, 2022, 85: 123-154. doi: 10.1016/j.semcancer.2021.05.010 33992782

[12] Liu F, Yang X, Geng M, et al. Targeting ERK, an Achilles’ Heel of the MAPK pathway, in cancer therapy. Acta Pharm Sin B, 2018, 8(4): 552-562. doi: 10.1016/j.apsb.2018.01.008 30109180 PMC6089851

[13] Frisone D, Friedlaender A, Malapelle U, et al. A BRAF new world. Crit Rev Oncol Hematol, 2020, 152: 103008. doi: 10.1016/j.critrevonc.2020.103008 32485528

[14] Davies H, Bignell GR, Cox C, et al. Mutations of the BRAF gene in human cancer. Nature, 2002, 417(6892): 949-954. doi: 10.1038/nature00766 12068308

[15] Halle BR, Johnson DB. Defining and targeting BRAF mutations in solid tumors. Curr Treat Options Oncol, 2021, 22(4): 30. doi: 10.1007/s11864-021-00827-2 33641072

[16] Cheng L, Lopez-Beltran A, Massari F, et al. Molecular testing for BRAF mutations to inform melanoma treatment decisions: a move toward precision medicine. Mod Pathol, 2018, 31(1): 24-38. doi: 10.1038/modpathol.2017.104 29148538 PMC5758899

[17] Stintzing S, Heinrich K, Tougeron D, et al. FOLFOXIRI plus Cetuximab or Bevacizumab as first-line treatment of BRAF(V600E)-mutant metastatic colorectal cancer: the randomized phase II FIRE-4.5 (AIO KRK0116) study. J Clin Oncol, 2023, 41(25): 4143-4153. doi: 10.1200/JCO.22.01420 37352476

[18] Sorbye H, Dragomir A, Sundstrom M, et al. High BRAF mutation frequency and marked survival differences in subgroups according to KRAS/BRAF mutation status and tumor tissue availability in a prospective population-based metastatic colorectal cancer cohort. PLoS One, 2015, 10(6): e0131046. doi: 10.1371/journal.pone.0131046 26121270 PMC4484806

[19] Califano I, Smulever A, Jerkovich F, et al. Advances in the management of anaplastic thyroid carcinoma: transforming a life-threatening condition into a potentially treatable disease. Rev Endocr Metab Disord, 2023. doi: 10.1007/s11154-023-09833-1 37648897

[20] Van den Bent MJ, Geurts M, French PJ, et al. Primary brain tumours in adults. Lancet, 2023, 402(10412): 1564-1579. doi: 10.1016/S0140-6736(23)01054-1 37738997

[21] Tang TY, Nichetti F, Kaplan B, et al. Comparative genomic analysis and clinical outcomes of BRAF-mutated advanced biliary tract cancers. Clin Cancer Res, 2023, 29(23): 4853-4862. doi: 10.1158/1078-0432.CCR-23-1926 37773629

[22] Acosta-Medina AA, Kemps PG, Zondag TCE, et al. BRAF V600E is associated with higher incidence of second cancers in adults with Langerhans cell histiocytosis. Blood, 2023, 142(18): 1570-1575. doi: 10.1182/blood.2023021212 37595284 PMC10797504

[23] Puri M, Gawri K, Dawar R. Therapeutic strategies for BRAF mutation in non-small cell lung cancer: a review. Front Oncol, 2023, 13: 1141876. doi: 10.3389/fonc.2023.1141876 37645429 PMC10461310

[24] Tabbo F, Pisano C, Mazieres J, et al. How far we have come targeting BRAF-mutant non-small cell lung cancer (NSCLC). Cancer Treat Rev, 2022, 103: 102335. doi: 10.1016/j.ctrv.2021.102335 35033867

[25] Mazieres J, Cropet C, Montane L, et al. Vemurafenib in non-small-cell lung cancer patients with BRAF(V600) and BRAF(nonV600) mutations. Ann Oncol, 2020, 31(2): 289-294. doi: 10.1016/j.annonc.2019.10.022 31959346

[26] Cai J, Jiang H, Li S, et al. The landscape of actionable genomic alterations by next-generation sequencing in tumor tissue versus circulating tumor DNA in Chinese patients with non-small cell lung cancer. Front Oncol, 2021, 11: 751106. doi: 10.3389/fonc.2021.751106 35273907 PMC8902245

[27] Ahn HY, Lee CH, Lee MK, et al. BRAF V600E mutation of non-small cell lung cancer in Korean patients. Medicina (Kaunas), 2023, 59(6): 1085. doi: 10.3390/medicina59061085 37374289 PMC10304407

[28] Lim GHT, Balbi KJ, Poskitt B, et al. Prevalence and breakdown of non-small cell lung cancer BRAF driver mutations in a large UK cohort. Lung Cancer, 2022, 173: 71-74. doi: 10.1016/j.lungcan.2022.09.008 36156323

[29] Overbeck TR, Reiffert A, Schmitz K, et al. NTRK gene fusions in non-small-cell lung cancer: real-world screening data of 1068 unselected patients. Cancers (Basel), 2023, 15(11): 2966. doi: 10.3390/cancers15112966 37296928 PMC10252111

[30] Cancer Genome Atlas Research Network. Comprehensive genomic characterization of squamous cell lung cancers. Nature, 2012, 489(7417): 519-525. doi: 10.1038/nature11404 22960745 PMC3466113

[31] Alrifai D, Popat S, Ahmed M, et al. A rare case of squamous cell carcinoma of the lung harbouring ALK and BRAF activating mutations. Lung Cancer, 2013, 80(3): 339-340. doi: 10.1016/j.lungcan.2013.02.002 23499398

[32] Marchetti A, Felicioni L, Malatesta S, et al. Clinical features and outcome of patients with non-small-cell lung cancer harboring BRAF mutations. J Clin Oncol, 2011, 29(26): 3574-3579. doi: 10.1200/JCO.2011.35.9638 21825258

[33] Litvak AM, Paik PK, Woo KM, et al. Clinical characteristics and course of 63 patients with BRAF mutant lung cancers. J Thorac Oncol, 2014, 9(11): 1669-1674. doi: 10.1097/JTO.0000000000000344 25436800 PMC4251710

[34] Khunger A, Khunger M, Velcheti V. Dabrafenib in combination with trametinib in the treatment of patients with BRAF V600-positive advanced or metastatic non-small cell lung cancer: clinical evidence and experience. Ther Adv Respir Dis, 2018, 12: 1753466618767611. doi: 10.1177/1753466618767611 29595366 PMC5941661

[35] McEvoy SH, Halpenny DF, Viteri-Jusue A, et al. Investigation of patterns of nodal metastases in BRAF mutant lung cancer. Lung Cancer, 2017, 108: 62-65. doi: 10.1016/j.lungcan.2017.02.024 28625649 PMC5538145

[36] Hwang I, Choi YL, Lee H, et al. Selection strategies and practical application of BRAF V600E-mutated non-small cell lung carcinoma. Cancer Res Treat, 2022, 54(3): 782-792. doi: 10.4143/crt.2021.843 34844291 PMC9296927

[37] Chu CH, Huang YH, Lee PH, et al. Various impacts of driver mutations on the PD-L 1 expression of NSCLC. PLoS One, 2022, 17(8): e0273207. doi: 10.1371/journal.pone.0273207 PMC9387808 35980949

[38] Dudnik E, Peled N, Nechushtan H, et al. BRAF mutant lung cancer: programmed death ligand 1 expression, tumor mutational burden, microsatellite instability status, and response to immune check-point inhibitors. J Thorac Oncol, 2018, 13(8): 1128-1137. doi: 10.1016/j.jtho.2018.04.024 29723688

[39] Li H, Zhang Y, Xu Y, et al. Tumor immune microenvironment and immunotherapy efficacy in BRAF mutation non-small-cell lung cancer. Cell Death Dis, 2022, 13(12): 1064. doi: 10.1038/s41419-022-05510-4 36543792 PMC9772302

[40] Sheikine Y, Pavlick D, Klempner SJ, et al. BRAF in lung cancers: Analysis of patient cases reveals recurrent BRAF mutations, fusions, kinase duplications, and concurrent alterations. JCO Precis Oncol, 2018, 2: PO.17.00172. doi: 10.1200/PO.17.00172 PMC7446447 32913992

[41] Dagogo-Jack I, Martinez P, Yeap BY, et al. Impact of BRAF mutation class on disease characteristics and clinical outcomes in BRAF-mutant lung cancer. Clin Cancer Res, 2019, 25(1): 158-165. doi: 10.1158/1078-0432.CCR-18-2062 30224342

[42] Aboubakar Nana F, Ocak S. Targeting BRAF activation as acquired resistance mechanism to EGFR tyrosine kinase inhibitors in EGFR-mutant non-small-cell lung cancer. Pharmaceutics, 2021, 13(9): 1478. doi: 10.3390/pharmaceutics13091478 34575554 PMC8471192

[43] Schaufler D, Ast DF, Tumbrink HL, et al. Clonal dynamics of BRAF-driven drug resistance in EGFR-mutant lung cancer. NPJ Precis Oncol, 2021, 5(1): 102. doi: 10.1038/s41698-021-00241-9 34921211 PMC8683498

[44] Dankner M, Rose AAN, Rajkumar S, et al. Classifying BRAF alterations in cancer: new rational therapeutic strategies for actionable mutations. Oncogene, 2018, 37(24): 3183-3199. doi: 10.1038/s41388-018-0171-x 29540830

[45] Barlesi F, Mazieres J, Merlio JP, et al. Routine molecular profiling of patients with advanced non-small-cell lung cancer: results of a 1-year nationwide programme of the French Cooperative Thoracic Intergroup (IFCT). Lancet, 2016, 387(10026): 1415-1426. doi: 10.1016/S0140-6736(16)00004-0 26777916

[46] Cardarella S, Ogino A, Nishino M, et al. Clinical, pathologic, and biologic features associated with BRAF mutations in non-small cell lung cancer. Clin Cancer Res, 2013, 19(16): 4532-4540. doi: 10.1158/1078-0432.CCR-13-0657 23833300 PMC3762878

[47] Couraud S, Barlesi F, Fontaine-Deraluelle C, et al. Clinical outcomes of non-small-cell lung cancer patients with BRAF mutations: results from the French Cooperative Thoracic Intergroup biomarkers France study. Eur J Cancer, 2019, 116: 86-97. doi: 10.1016/j.ejca.2019.04.016 31181537

[48] Guisier F, Dubos-Arvis C, Vinas F, et al. Efficacy and safety of anti-PD-1 immunotherapy in patients with advanced NSCLC with BRAF, HER2, or MET mutations or RET translocation: GFPC 01-2018. J Thorac Oncol, 2020, 15(4): 628-636. doi: 10.1016/j.jtho.2019.12.129 31945494

[49] Niu X, Sun Y, Planchard D, et al. Durable response to the combination of Atezolizumab with Platinum-based chemotherapy in an untreated non-smoking lung adenocarcinoma patient with BRAF V600E mutation: A case report. Front Oncol, 2021, 11: 634920. doi: 10.3389/fonc.2021.634920 34178624 PMC8222507

[50] Lung Cancer Professional Committee of China Anti-Cancer Association. Expert consensus on the diagnosis and treatment of BRAF mutations in advanced non-small cell lung cancer in China. Zhonghua Zhongliu Zazhi, 2023, 45(4): 279-290. 37078209 10.3760/cma.j.cn112152-20230117-00030

[51] Planchard D, Kim TM, Mazieres J, et al. Dabrafenib in patients with BRAF (V600E)-positive advanced non-small-cell lung cancer: a single-arm, multicentre, open-label, phase 2 trial. Lancet Oncol, 2016, 17(5): 642-650. doi: 10.1016/S1470- 2045(16)00077-2 10.1016/S1470-2045(16)00077-2 PMC5006181 27080216

[52] Subbiah V, Gervais R, Riely G, et al. Efficacy of Vemurafenib in patients with non-small-cell lung cancer with BRAF V600 mutation: An open-label, single-arm cohort of the histology-independent VE-BASKET study. JCO Precis Oncol, 2019, 3: PO.18.00266. doi: 10.1200/PO.18.00266 PMC7446432 32914022

[53] Planchard D, Smit EF, Groen HJM, et al. Dabrafenib plus trametinib in patients with previously untreated BRAF (V600E)-mutant metastatic non-small-cell lung cancer: an open-label, phase 2 trial. Lancet Oncol, 2017, 18(10): 1307-1316. doi: 10.1016/S1470- 2045(17)30679-4 10.1016/S1470-2045(17)30679-4 28919011

[54] Li J, Sasane M, Zhang J, et al. Is time to progression associated with post-progression survival in previously treated metastatic non-small cell lung cancer with BRAF V600E mutation? A secondary analysis of phase II clinical trial data. BMJ Open, 2018, 8(8): e021642. doi: 10.1136/bmjopen-2018-021642 PMC6104743 30121602

[55] Planchard D, Besse B, Groen HJM, et al. Phase 2 study of Dabrafenib plus Trametinib in patients with BRAF V600E-mutant metastatic NSCLC: Updated 5-year survival rates and genomic analysis. J Thorac Oncol, 2022, 17(1): 103-115. doi: 10.1016/j.jtho.2021.08.011 34455067

[56] Guidelines Working Committee of the Chinese Clinical Oncology Society. Chinese Society of Clinical Oncology (CSCO) guidelines for the diagnosis and treatment of non-small cell lung cancer, 2023. Beijing: People’s Health Publishing House, 2023.

[57] Riely GJ, Smit EF, Ahn MJ, et al. Phase II, open-label study of Encorafenib plus Binimetinib in patients with BRAF(V600)-mutant metastatic non-small-cell lung cancer. J Clin Oncol, 2023, 41(21): 3700-3711. doi: 10.1200/JCO.23.00774 37270692

[58] Planchard D, Besse B, Groen HJM, et al. Dabrafenib plus trametinib in patients with previously treated BRAF (V600E)-mutant metastatic non-small cell lung cancer: an open-label, multicentre phase 2 trial. Lancet Oncol, 2016, 17(7): 984-993. doi: 10.1016/S1470-2045(16)30146-2 27283860 PMC4993103

[59] Schadendorf D, Robert C, Dummer R, et al. Pyrexia in patients treated with dabrafenib plus trametinib across clinical trials in BRAF-mutant cancers. Eur J Cancer, 2021, 153: 234-241. doi: 10.1016/j.ejca.2021.05.005 34225229

[60] Atkinson V, Long GV, Menzies AM, et al. Optimizing combination dabrafenib and trametinib therapy in BRAF mutation-positive advanced melanoma patients: Guidelines from Australian melanoma medical oncologists. Asia Pac J Clin Oncol, 2016, 12(Suppl 7): 5-12. doi: 10.1111/ajco.12656 27905182

[61] Subbiah V, Baik C, Kirkwood JM. Clinical development of BRAF plus MEK inhibitor combinations. Trends Cancer, 2020, 6(9): 797-810. doi: 10.1016/j.trecan.2020.05.009 32540454

[62] Rudin CM, Hong K, Streit M. Molecular characterization of acquired resistance to the BRAF inhibitor dabrafenib in a patient with BRAF-mutant non-small-cell lung cancer. J Thorac Oncol, 2013, 8(5): e41-e42. doi: 10.1097/JTO.0b013e31828bb1b3 23524406 PMC3634121

[63] Poulikakos PI, Rosen N. Mutant BRAF melanomas--dependence and resistance. Cancer Cell, 2011, 19(1): 11-15. doi: 10.1016/j.ccr.2011.01.008 21251612

[64] Oddo D, Sennott EM, Barault L, et al. Molecular landscape of acquired resistance to targeted therapy combinations in BRAF-mutant colorectal cancer. Cancer Res, 2016, 76(15): 4504-4515. doi: 10.1158/0008-5472.CAN-16-0396 27312529 PMC4970882

[65] Huo KG, Notsuda H, Fang Z, et al. Lung cancer driven by BRAF(G469V) mutation is targetable by EGFR kinase inhibitors. J Thorac Oncol, 2022, 17(2): 277-288. doi: 10.1016/j.jtho.2021.09.008 34648945

[66] Yao Z, Gao Y, Su W, et al. RAF inhibitor PLX8394 selectively disrupts BRAF dimers and RAS-independent BRAF-mutant-driven signaling. Nat Med, 2019, 25(2): 284-291. doi: 10.1038/s41591-018-0274-5 30559419 PMC6404779

